# Midwife Led Units: Transforming Maternity Care Globally

**DOI:** 10.5334/aogh.2794

**Published:** 2020-04-28

**Authors:** Joyce K. Edmonds, Juliana Ivanof, Ursula Kafulafula

**Affiliations:** 1 Boston College School of Nursing, US; 2 Kamuzu College of Nursing, MW

## Abstract

**Background:**

Midwifery-led care is a high-certainty, evidence-based strategy to improve maternity care. Midwife-led units (MLUs) are one example of how the midwifery model of care is being integrated into existing health systems to transform maternal health around the world.

**Purpose:**

To promote global investment in MLUs by describing the benefits, current advances and future directions of this model of care.

**Method:**

A viewpoint based on prevalent notions of midwifery, research findings, guidance from professional organizations and authors’ professional experience.

**Conclusion:**

Renewed commitment to research and the implementation of MLUs across a variety of settings is needed to address the practice, education and policy issues associated with this evidence-based strategy. The World Health Organization “Year of the Nurse and Midwife-2020” is an opportune time to invest in midwifery models of care that are fundamental to achieving core global health initiatives such as Universal Healthcare 2030.

The World Health Organization (WHO) designated 2020 as the “Year of the Nurse and Midwife”, an honor intended to draw global attention to the essential role nurses and midwives have in transforming healthcare [1]. Midwives are coming together around the world to change how maternity care is organized and delivered and in turn improving women’s experiences and outcomes in childbirth. Maternity care refers to the healthcare provided to women during pregnancy, childbirth and the postpartum period, and vast advances are required to improve maternal and newborn survival, reduce high rates of maternal and neonatal morbidity, curb over-medicalization of care, and ensure dignity and respect in pregnancy and childbirth. In this paper, we discuss midwifery led units (MLUs) in the global maternal health context as a strategic way to offer women-centered care and maximize the health outcomes of women and infants.

Midwifery-led care is a high-certainty, evidence-based strategy to improve maternity care. The universal philosophy of midwives emphasizes care that promotes normal physiologic pregnancy and labor and supports the natural ability of women to experience birth with minimum or no routine intervention. Midwives practice holistic care guided by the ethical principles of justice, equity, and respect for human dignity, and their practice is grounded in continuous education and the use of scientific research and evidence [2]. The International Confederation of Midwives (ICM) offers standards for rigorous, competency-based education to ensure that midwives are able to provide optimal care, and therefore posit that midwives “are the professionals of choice for childbearing women in all areas of the world” [3]. A recent WHO report outlines a seven-step action plan to strengthen the quality of midwifery education to these international standards [4]. Ensuring that all midwives have core competencies for practice is essential to realizing three global health initiatives: WHO’s Global Strategy for Women’s, Children’s and Adolescent’s Health 2016–2030, the Sustainable Development Goals and Universal Health Care Coverage by 2030 [5].

There are an estimated 1.1 million midwives documented globally and far more who provide care to women and their families but are not officially counted [6]. Lack of data makes the enumeration of midwives and their models of care challenging. However, the accumulated body of evidence demonstrates that maternity care involving a midwife as the main care provider leads to several positive outcomes with no adverse effects for both mothers and their babies [7]. In fact, midwifery care for women at low-risk for complications during pregnancy is associated with various benefits, such as increased rates of maternal satisfaction and a decrease in unnecessary medical interventions [8]. This robust evidence base led the National Institute for Health and Care Excellence (NICE) to recommend midwifery-led settings as the safest birthplace for healthy women experiencing uncomplicated pregnancies in the United Kingdom [9].

In addition to improving maternal and newborn health outcomes, the evidence shows that better integration of midwives into health systems is fundamental to reducing primary and maternity care provider shortages and to addressing racial, ethnic and geographic health inequality [10]. Midwives often provide care to the most socially and economic vulnerable women in fragile settings and offer more than just intrapartum care. In fact, quality midwifery care improves over 50 health-related outcomes in areas such as breastfeeding, cancer and cardiometabolic disease prevention, tobacco cessation, sexual and reproductive health as well as early childhood development [11]. Outcomes impacted by midwifery care have a positive influence on the Sustainable Development Goals (SDGs) and are fundamental to achievement of Universal Healthcare.

Specifically, midwife-led units (MLUs) are one exemplar of how the midwifery model of care is being integrated into existing health systems to transform maternal health. The MLU is a relatively “new” care model in which the midwife is the primary healthcare professional caring for low-risk pregnant women, as opposed to being cared for by a medical team headed by an obstetrician or consultant [12]. Accordingly, MLUs also provide a space within which midwives can practice to their fullest potential with more professional autonomy than in a traditional obstetric setting [13]. There are two main types of MLUs, either alongside (also known as onsite midwife led birth units) or freestanding (also known as stand-alone), both of which may be referred to as “birth centers” in the literature [14]. Alongside midwifery units (AMU) co-exist in the same building or in a separate building on the same site as a hospital or host obstetric unit [15]. In the event a laboring women needs comprehensive emergency obstetric care, she can be transferred via walking, wheelchair or by rolling bed or trolley. AMUs are thought to provide the desirable attributes of both home and hospital, offering biopsychosocial safety in a welcoming environment with care, characteristic of the midwifery model. In contrast, freestanding midwifery units are geographically separate from the hospital obstetric unit and women must transfer via ambulance or vehicle in the event of complications in labor [12]. For low-risk pregnancies, there is evidence that care in freestanding birth centers results in equivalent or better outcomes than hospital-based care in the United States [16].

Midwifery-led units can be conceived as a response to the phenomenon coined by The Lancet’s Maternal Health Series as “too much, too soon,” which refers to care before, during and after childbirth that is too much, unnecessary, inappropriate, and possibly even harmful [17]. It is one extreme in maternity care, with “too little, too late” at the other extreme. MLUs promote women-centered care, choice, control and continuity of care, and from a feminist perspective counter the prevailing culture of care in specialized hospitals where a high level of technological and medicalized birth practices, emblematic of patriarchy, is valued [18]. Anecdotal evidence suggests that grassroots efforts by midwives, who have witnessed the widespread abuse and disrespect of women in obstetric facilities and lack of evidence-based care, are driving the move toward MLUs in hospitals.

In high-income countries, MLUs offer a respite from the medicalization of childbirth that has gradually worsened, such as in Switzerland, where over 30% of births are now delivered by cesarean. A 2015 qualitative study conducted at a Swiss hospital examined women and providers’ perceptions of a proposed MLU, and the results were generally positive. Women who had previously delivered in the hospital’s obstetrician-led unit were particularly interested in the normalization of childbirth that midwife-led care provided, as well as the continuity of care throughout their pregnancy and childbirth process [19]. The midwives and obstetricians interviewed were also in favor of developing an MLU as an opportunity to bring about positive change [19]. Countries such as Switzerland can draw from examples of successful MLUs in places such as Malawi. A performance and quality improvement study performed there demonstrated that despite few resources and physicians, the quality of care provided by midwives was high, particularly in antenatal care and labor and delivery [20]. In Malawi, midwives are typically the healthcare professional caring for pregnant women. As in many low-income countries, midwife-led care is the norm or provided by default in the absence of obstetricians rather than by design.

However, effective midwifery training programs, especially in low-resource settings such as Malawi, are hampered by lack of resources, student exposure to unsafe practices, and absence of faculty control over the practice environment. There is a theory-to-practice gap between what students learn during their midwifery training and what they experience in clinical practice. Theoretical information about the essence of midwifery care given in lectures is not modeled by midwives in clinical practice and the student experience greatly suffers. This theory-to-practice gap leads to graduating midwives who are less competent to care for women and their newborn babies than is ideal. Kamuzu College of Nursing (KCN), a constituent college of the University of Malawi, in collaboration with Queen Elizabeth Central Hospital (QECH), the largest referral and teaching hospital in Malawi, endeavour to be canters of excellence in midwifery education and practice. Currently, QECH is working on a proposal to institute an MLU to improve the quality of clinical education and women-centered practice at Queen Elizabeth Hospital by 2024. To-date a series of consultative meetings with stakeholders including obstetricians working at QECH have been held. QECH has provided space within its existing infrastructure for the MLU and a memorandum of understanding has been signed between the university and hospital. With financial assistance from Seed Global Health, a midwifery educator has been in place since July 2019 to assist with establishing the ward. Clinical guidelines, policies and procedures for the ward are complete.

The effects of MLUs in improving health outcomes has predominately based on research in high-income countries, such as England [14], Australia [21], Switzerland [18], Ireland [22], and Japan [23] and are viewed as way to promote women’s choice of place of birth and reduce unnecessary intervention in childbirth. A more limited body of research from low- and middle- income countries such as South Africa [24] and Malawi [20] have documented midwifery units as a means to assure women access to respectful, high-quality care although the model is not standardized [25].

There is a critical need to examine MLUs in low- and middle-income countries where 99% of global maternal and neonatal deaths continue to occur. Notably, the global shift from home to facility-based care, that drove the relative success of Millennium Development Goal 5, had the unintended consequence of increasing unnecessary and costly interventions beyond the amount needed to reduce mortality. MLUs offer a middle path by reducing the likelihood of potentially harmful interventions while providing access to higher level emergency obstetric care, a fitting option for women who enter childbirth at low risk of complications and for health systems aiming to improve the quality, cost and experiences of maternity care. It is thought that AMUs can help meet the growing demand for facility-based birth and might be particularly beneficial in settings where universal access to higher level facility-based care is limited [26].

MLUs, such as the one being developed in Malawi, need continued exploration and evaluation to assess the impact on outcomes, quality and costs. Implementation research into how to design, scale and sustain MLUs including the cultural and social contexts that best enable success will be critical for improving MLUs. The Midwifery Units Networking Project at the Centre for Maternal and Child Health Research at University of London, for example, is working on facilitating research that addresses MLUs in low- and middle-income countries. In addition, data on the configuration of midwifery units (types, numbers, and utilization), similar to the mapping of midwifery units in England by the Birthplace in England Research Program, will help inform global maternity care policy. Nationally representative data shows that births in MLUs in England have increased from 5 percent to 14 percent and the number of AMUs almost doubled from 53 to 97 during a six-year period between 2010 to 2016. Currently, population level data to evaluate the availability and utilization of MLUs is limited to select high-income countries where midwifery is well established [27].

Every woman, everywhere has a right to respectful, high-quality care in childbirth. The preservation of this basic right is at the essence of midwifery care. Midwifery models of care should be prioritized for funding and supported by regulatory legislation that provides adequate educational infrastructure and recognizes midwifery as an autonomous health profession, actualizing WHO’s action plan for strengthening quality midwifery education [5]. The WHO designation of 2020 as the “Year of the Nurse and Midwife” brings recognition to the central role of midwives and serves as a catalyst to accelerate financial investment in midwifery and midwifery models of care that will bring improvements to maternal healthcare and beyond.

We call for the global community to respond and place midwifery high on the global health agenda.
